# Changes in cognition, coping, pain and emotions after 12-months access to the digital self-management program EPIO

**DOI:** 10.3389/fpsyg.2025.1540852

**Published:** 2025-02-25

**Authors:** Elin Bolle Strand, Cecilie Varsi, Elin Børøsund, Hilde Eide, Karlein M. G. Schreurs, Lori B. Waxenberg, Karen E. Weiss, Eleshia J. Morrison, Hanne Stavenes Støle, Ólöf Birna Kristjansdottir, Audun Stubhaug, Lise Solberg Nes

**Affiliations:** ^1^Department of Digital Health Research, Division of Medicine, Oslo University Hospital, Oslo, Norway; ^2^Faculty of Health Sciences, VID Specialized University, Oslo, Norway; ^3^Faculty of Health and Social Sciences, University of South-Eastern Norway, Drammen, Norway; ^4^Department of Nursing and Health Sciences, Faculty of Health and Social Sciences, University of South-Eastern Norway, Drammen, Norway; ^5^Centre for Health and Technology, Faculty of Health and Social Sciences, University of South-Eastern Norway, Drammen, Norway; ^6^Department of Psychology, Health & Technology, University of Twente, Enschede, Netherlands; ^7^Department of Clinical and Health Psychology, University of Florida, Gainesville, FL, United States; ^8^Department of Psychiatry and Psychology, College of Medicine and Science, Mayo Clinic, Rochester, MN, United States; ^9^Department of Child Health and Development, Norwegian Institute of Public Health, Oslo, Norway; ^10^Mental Health Team West, Primary Care of the Capital Area, Reykjavik, Iceland; ^11^Department of Pain Management and Research, Oslo University Hospital, Oslo, Norway; ^12^Regional Advisory of Pain, University of Oslo, Oslo, Norway; ^13^Institute of Clinical Medicine, Faculty of Medicine, University of Oslo, Oslo, Norway

**Keywords:** digital, pain acceptance, pain reduction, emotion regulation, qualitative research

## Abstract

**Background:**

Psychosocial pain self-management interventions can be of support for people living with chronic pain. Since psychosocial support is not always accessible, digital health interventions may increase outreach of these types of evidence-based interventions.

**Objectives:**

To explore participants' experiences from 12-month access to the digital pain self-management program EPIO, particularly in terms of any behavioral and/or psychological changes experienced.

**Methods:**

Participants (*N* = 25) engaged in individual semi-structured interviews following 12-month access to the EPIO intervention. Qualitative thematic analyses were conducted seeking to identify any behavioral and/or psychological changes experienced through intervention use, and what contributed to these changes.

**Results:**

Participants were predominantly women (72%), median age 46 (range 26–70), with a range of self-reported pain conditions and the majority reporting pain duration >10 years (64%). Analyses identified three main themes and subsequent sub-themes: (1) *Changes in Cognition*; insight and self-awareness, acceptance and shifting focus, (2) *Changes in Coping*; pain, emotions, and activity pacing, and (3) *Content and Functionality Specific Engagement*; breathing and other mind-body exercises, thought-reflection exercises, and functionalities.

**Conclusions:**

People with chronic pain experienced positive behavioral and/or psychological changes in terms of cognition and coping after 12 months access to the EPIO digital pain self-management program. The most prominent changes included increased understanding of the connection between own thoughts, feelings, and behavior, gaining concrete strategies to cope with everyday life living with pain, and utilizing these strategies to reduce pain and interference of pain, as well as to improve emotion regulation and psychological wellbeing.

## 1 Introduction

Living with chronic pain involves a wide range of challenges for those impacted. In addition to the actual pain and subsequent physical limitations, chronic pain affects psychological health and wellbeing, physical and social activities, capacity to self-regulate, personal and professional roles, and disrupts necessary health behaviors such as sleep and physical activity (Cohen et al., 2021; Dansie and Turk, 2013; Mills et al., 2019; Solberg Nes et al., 2009). The multifaceted impact of pain has brought along a recognition of the biopsychosocial aspects of pain (Cohen et al., 2021), accompanied by recommendations for biopsychosocial treatment approaches for people living with chronic pain, including the well-known psychosocial self-management treatment approach of Cognitive Behavioral Therapy (CBT: Beck, 2020; Feliu-Soler et al., 2018; Williams et al., 2020), and more recent approaches such as Acceptance and Commitment Therapy (ACT; Driscoll et al., 2021; Feliu-Soler et al., 2018; Hayes, 2004; Joypaul et al., 2019).

Psychosocial pain self-management approaches such as CBT and ACT are usually delivered in either individual- or group therapy settings, which entail known delivery barriers including inadequate outreach and availability (Driscoll et al., 2021). In response to these challenges, digital health solutions are rapidly emerging with the aim of improving outreach of effective pain self-management interventions, some of which have shown promising results in terms of reduced pain intensity as well as improved mental health and quality of life (Moman et al., 2019). However, challenges with digital pain self-management interventions so far include: a need for evidence-based content (Devan et al., 2019; Zhao et al., 2019), limited end-user and healthcare provider input during design and development (Talboom-Kamp et al., 2018), significant problems related to study/intervention attrition (Amagai et al., 2022; Ludden et al., 2015), limited evidence of intervention efficacy both in the short and particularly long-term (Lee et al., 2018; Moman et al., 2019; Thurnheer et al., 2018), and limited intervention implementation into actual practice post study (Varsi et al., 2019).

Considering these shortcomings with existing digital self-management interventions, the current research team designed and developed a digital pain self-management intervention informed by evidence, with extensive stakeholder involvement and called EPIO, inspired by the Greek goddess for the soothing of pain; Epione (Bostrom et al., 2020; Bostrøm et al., 2022; Ledel Solem et al., 2020, 2019; Varsi et al., 2021). The EPIO intervention program was subsequently tested in a feasibility pilot study, with quantitative and qualitative examinations (O'Cathain et al., 2013; Richards et al., 2019) prior to program optimization in preparation for efficacy testing in a randomized controlled trial (RCT; (Skivington et al., 2021). Feasibility findings showed how participants found EPIO to be useful and easy to use, with excellent user satisfaction (Bostrom et al., 2020). Supplementing qualitative findings from post feasibility study interviews exploring participants' experiences when engaging with the digital program revealed themes of EPIO fostering joy and enthusiasm and raising awareness, with participants perceiving EPIO as a friend, and making peace with the presence of pain (Bostrom et al., 2020).

Following optimization (Bostrom et al., 2020), the EPIO intervention was tested over 12 months in an RCT, with findings showing statistically significant results in favor of the intervention group after three (i.e., reduced symptoms of depression and self-regulatory fatigue compared to controls (Bostrøm et al., 2023) and 12 months, with reduced symptoms of anxiety and depression, reduced self-regulatory fatigue and pain catastrophizing, and improved health-related quality of life (HRQoL), for participants having access to the EPIO intervention over 12 months compared with usual care controls (Solberg Nes et al., 2024).

Seeking to investigate what might have contributed to these changes, and whether any additional changes not captured by the quantitative analysis could be identified (O'Cathain et al., 2013; Richards et al., 2019), the current qualitative study aimed to further explore participants' experiences from having access to the EPIO intervention program over 12 months, particularly in terms of any behavioral and/or psychological changes experienced.

## 2 Materials and methods

### 2.1 Study design

This qualitative exploratory study presents insights derived from individual interviews with people with various chronic pain conditions after 12-month participation in an RCT evaluating the efficacy of the EPIO pain self-management program (Solberg Nes et al., 2024). The overall inclusion criteria for the RCT were: (1) Living with chronic pain (i.e., not pain condition/diagnosis-specific); (2) Having lived with pain ≥ 3 months; (3) Being ≥ 18 years old; (4) Having access to a smartphone or tablet; (5) Being able to understand oral and written Norwegian; and (5) Being able to attend an introduction session either at a health care facility or through a secure video link (i.e., due to Covid-19 pandemic restrictions as of spring 2020). Exclusion criteria included self-reported cancer-related pain, migraine or severe untreated psychological illness (e.g., psychosis).

Supplementary inclusion criteria for the current study stipulated that participants were assigned to the intervention group of the RCT (i.e., had been granted access to the EPIO program for the duration of 12 months), had completed the 12-month outcome measures, and expressed willingness to engage in an individual interview after 12 months study completion. To ensure sample heterogeneity, a recruitment matrix was created based on the RCT sample (Solberg Nes et al., 2024) and utilized to strive for balanced representation across gender, age, years lived with pain, type of introduction session, and program progress in the current study. Potential selection impact (i.e., RCT population vs. qualitative sample) is addressed in the Discussion section.

### 2.2 The EPIO intervention program

**Figure 1:**
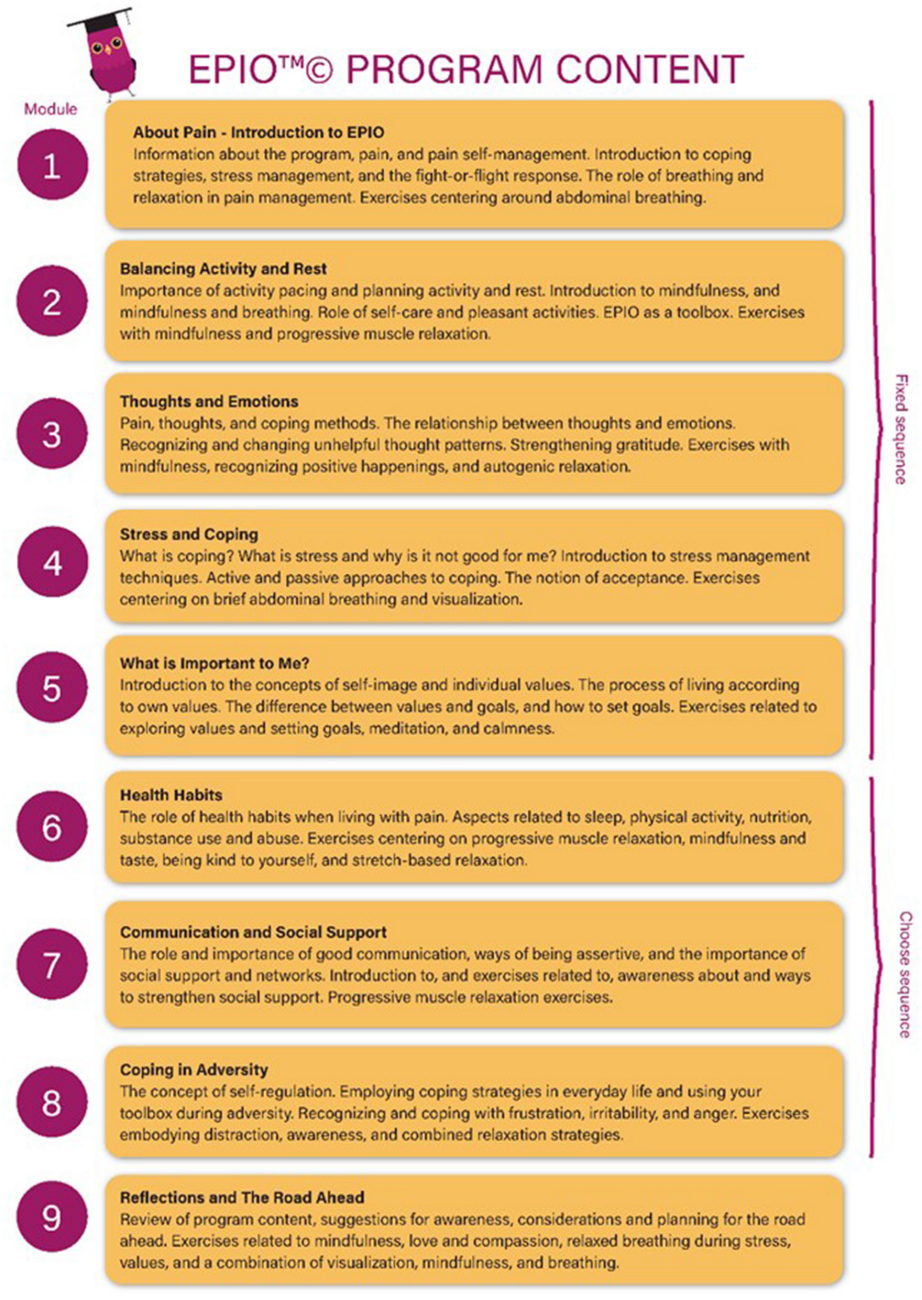
Overview EPIO program modules and content.

The EPIO program is delivered through an application (app) and consists of nine modules with user-centered, evidence-informed design and content. The content is primarily CBT-based, with incorporated aspects of ACT. Each module contains a combination of psychoeducational content, recommended strategies for pain self-management, and exercises (e.g., diaphragmatic breathing, thought challenging, mindfulness, visualization: Ledel Solem et al., 2020). See Figure 1 for an overview of EPIO modules and content.

**Figure 2:**
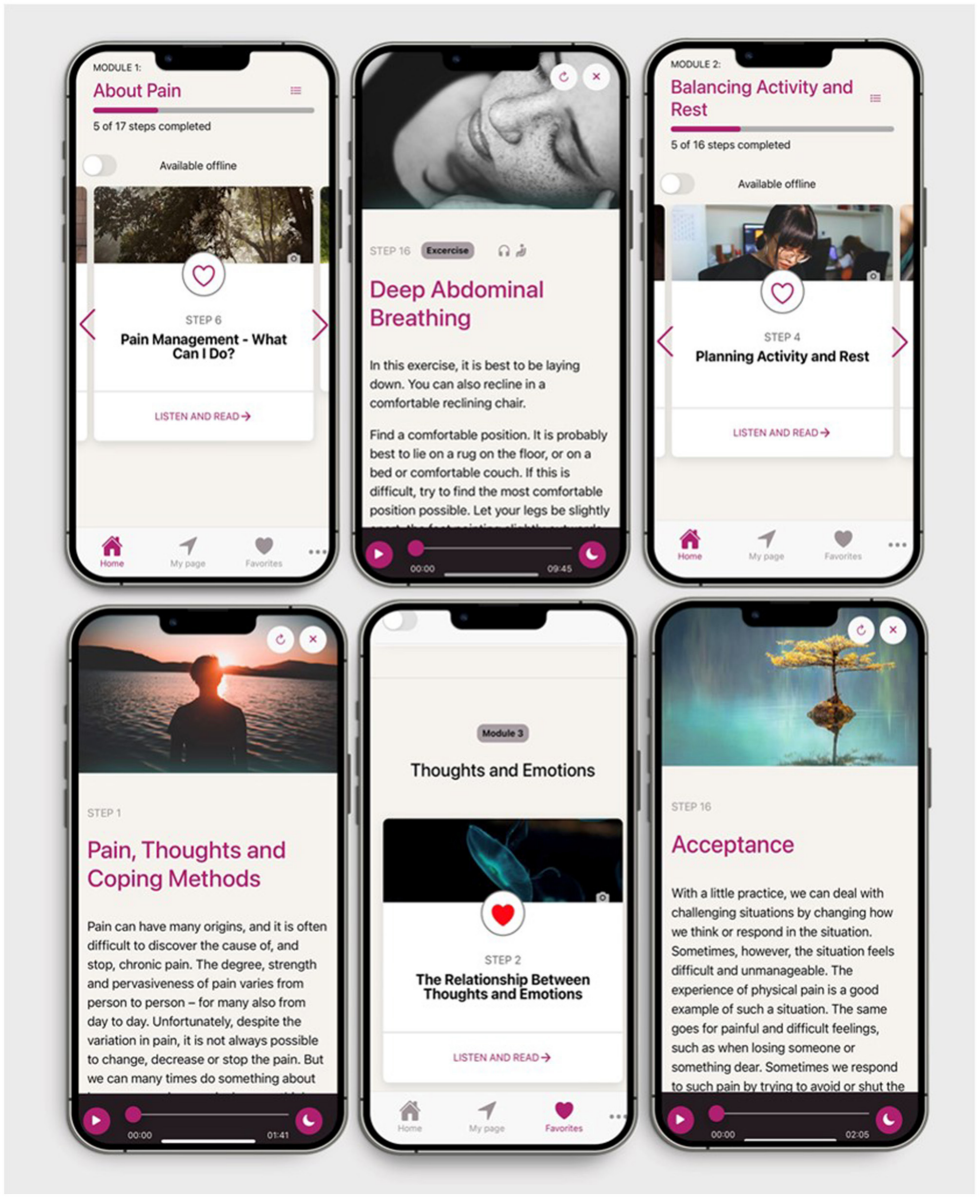
EPIO program example screenshots.

The EPIO program includes functionalities such as stepwise presentations within each of the nine modules, with the avatar EPIOS (i.e., an animated bird) providing summaries and reminders throughout. The first five modules are considered building blocks for each other and consecutively presented, while the sequence of module six to eight can be individually chosen (see Figure 1). To ensure personalization, an overview of personal registrations (i.e., sleep, rest, activity level, mood), program progress, and achieved progress trophies are included in a *My page* feature. Also, a *My favorites* option allows users to mark favorite content or sections to allow for easy access to favorite aspects. The EPIO program also allows users to choose between reading or listening, and to receive program reminders. With CBT and ACT encouraging “homework” and practice in-between sessions, the EPIO program contains a 3-day delay between the modules to encourage practice. The rationale being that taking time to practice and becoming familiar with the content, rather than “rushing” ahead, hopefully would be of benefit. Please see Figure 2 for examples of EPIO screenshots.

As studes involving guided digital interventions have shown stronger effect compared to self-guided interventions, and recommendations for blended care intervention delivery have surfaced (Aerts and van Dam, 2018; Obro et al., 2021; Talboom-Kamp et al., 2018), the EPIO intervention was delivered in a simple blended care delivery model in the feasibility pilot study as well as in the RCT (Bostrom et al., 2020; Solberg Nes et al., 2024), with one introduction session, the nine within app modules, and two follow-up phone calls at approximately three and seven weeks post introduction session.

### 2.3 Data collection

A semi-structured interview guide was developed through a synthesis of existing literature related to people's experiences with digital interventions for chronic pain and drawing upon the research team's insights gained from design, development, feasibility and efficacy testing of EPIO. The interview guide contained questions about participants' experiences while utilizing EPIO over the 12 months, how they had fared during this time, any behavioral and/or psychological changes they might have experienced during the use of EPIO, and which specific aspects of EPIO, if any, contributed to such changes. The individual interviews were conducted by one of three EPIO project team members who were public health scientists and/or registered nurses, trained and supervised by the principal investigator who is a licensed clinical health psychologist. The interviews were conducted via telephone, lasted median 40 min (range 23 to 70 min), were audio-recorded and transcribed verbatim.

### 2.4 Analyses

The analyses were guided by thematic analysis with the six-step approach as outlined by Braun and Clarke (2006). The initial analysis of the transcribed interviews was carried out by the first (EBS) and second author (CV), with the intention of extracting data items and change-related codes for further analyses, after familiarizing themselves with the data (i.e., step 1). Step 2 derived codes from the data, identifying and extracting entrenched meaning from the transcribed text (e.g., “self-awareness” extracted from units such as “*Using EPIO has opened up awareness around thoughts, feelings and actions*”). In step 3, codes were combined in sub-themes that were iteratively discussed between the first and second author. With the aim of constructing overall themes and to ensure consistency between results and the focus of the study, codes and sub-themes were further discussed (step 4 and 5) in the core research team (EBS, CV, LSN, EB). Next, the main themes were presented, together with sub-themes, codes and core team reflections, and discussed with all co-authors in terms of name, content, and meaning. Finally, illustrative quotes from the interviews were selected to legitimize and ensure credibility and transparency of the analyses, and the write up of the results was then competed (step 6).

### 2.5 Ethical approval

The current project has been approved by the Regional Ethical Committee for Medical and Health Research Ethics (REK 2018/8911) and the Oslo University Hospital Institutional Review Board equivalent (PVO 2017/6697). All participants provided written informed consent prior to study inclusion.

## 3 Results

### 3.1 Study participants

A total of 36 participants who had initially expressed interest in being interviewed were contacted via telephone or text message after 12-month program access, of which 25 agreed to be interviewed. Since data analysis indicated that this number of participants provided sufficient information power (saturation) of the data material (Malterud et al., 2016), no additional interviews were necessary. The matrix used for study inclusion ensured a variability in participant characteristics, even though the majority of participants interviewed were female, married/living with a partner, on sick leave/disability benefits, had primary musculoskeletal pain (e.g., Fibromyalgia, unspecified musculoskeletal pain; Nicholas et al., 2019), had lived with pain for more than 10 years, and had completed all EPIO modules. Average number of days accessing the program was 37, with some using the program less, and others much more. Please see Table 1 for participant demographic details.

**Table 1:** Socio-demographic and disease-related participant characteristics.

**Characteristics**	**Participants in qualitative interviews (*N* = 25)**
Age (years), median (range)	46 (26–70)
**Gender**, ***n*** **(%)**	
Female	18 (72)
Male	7 (28)
Married/living with a partner, *n* (%)	18 (72)
**Employment**, ***n*** **(%)**	
Full time/part time	7 (28)
Sick leave/disability benefits	15 (60)
Retired/other	3 (12)
Pain conditions^*^, ***n*** **(%)**	
Rheumatoid arthritis	5 (20)
Osteoarthritis	4 (16)
Fibromyalgia	9 (36)
Unspecific musculoskeletal pain	7 (28)
Unspecified disc disorder	6 (24)
Neuropathic pain	1 (4)
Post-injury/surgery	1 (4)
Other	3 (12)
**Pain duration**, ***n*** **(%)**	
Low (< 3 years)	3 (12)
Moderate (3–10 years)	6 (24)
High (>10 years)	16 (64)
**Module completion**, ***n*** **(%)**	
Low (1–2 modules completed)	3 (12)
Moderate (3–6 modules completed)	4 (16)
High (7–9 modules completed)	18 (72)
Days accessing the program, median (range)	37 (5–315)
**Introduction session type**, ***n*** **(%)**	
In-person	12 (48)
Video	13 (52)

^*^Participants could report having several types of pain conditions.

**Figure 3:**
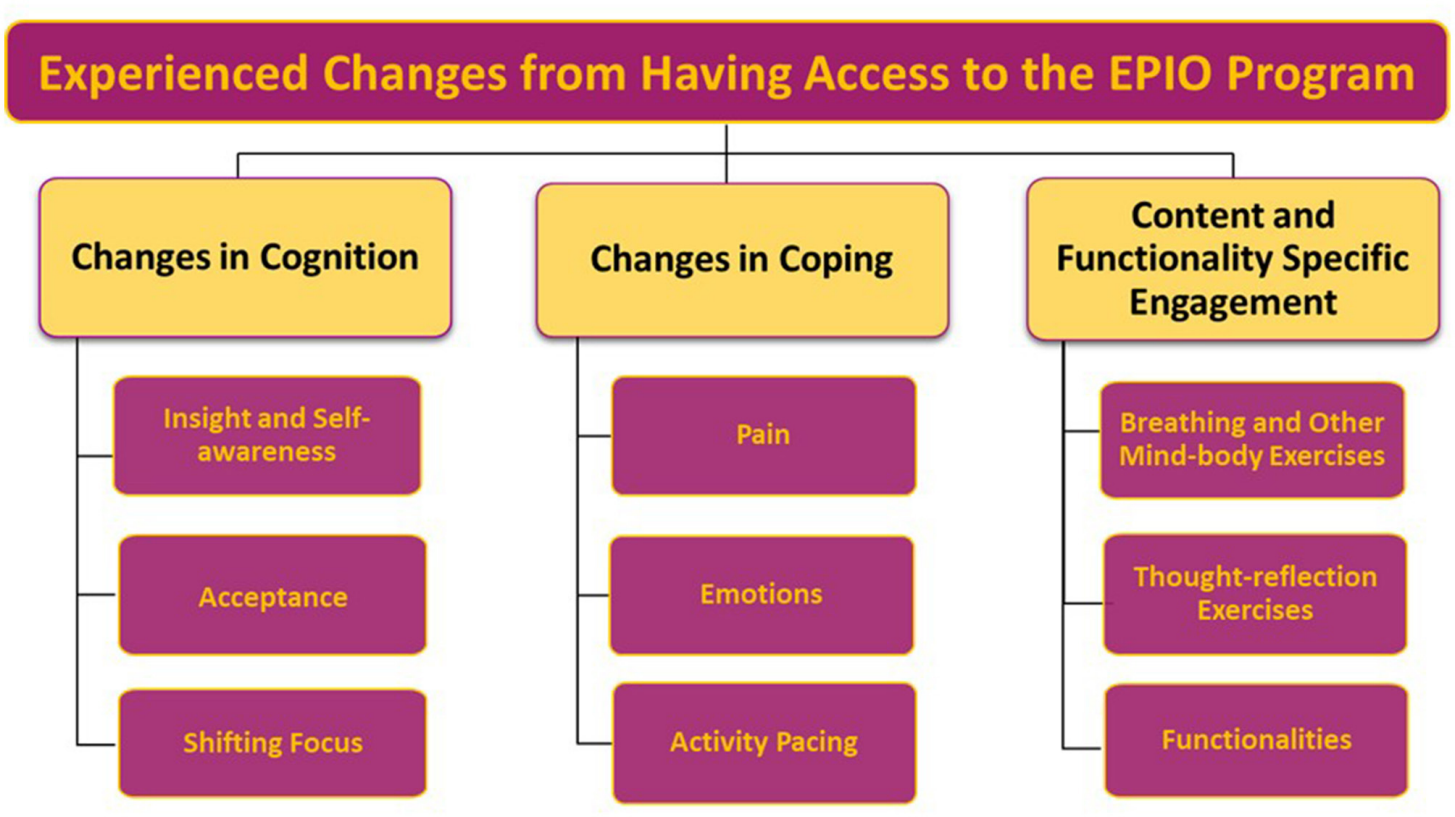
Overview of identified main themes and sub-themes related to participants' experienced changes from having access to the EPIO intervention program.

The qualitative analyses identified aspects related to experienced changes and any other impact from having access to the EPIO intervention program, and findings were organized into three main themes: (1) *Changes in Cognition, (*2) *Changes in Coping*, and (3) *Content and Functionality Specific Engagement*. Within each of the three main themes, several sub-themes were also identified. Please see Figure 3 for an illustration of identified themes and sub-themes related to experienced changes from having access to EPIO.

### 3.2 Changes in cognition

The participants described the EPIO program as creating a realization of how their thoughts can impact feelings and behavior, creating a new comprehension and way of thinking about life with pain. They referred to these new ways of thinking as also inducing acceptance, allowing for better ways to live *with* pain, as well as focusing more on positive rather than negative thoughts as a consequence of using the program. Themes surfacing related to changes in cognition were sorted into three sub-themes: Insight and self-awareness, acceptance, and shifting focus.

#### 3.2.1 Sub-theme 1: insight and self-awareness

Several participants reported having become more aware of the connection between their own thoughts and emotions and how they approached various situations after becoming acquainted with EPIO. They described increased awareness of how thoughts can be crucial for how you feel, and having realized how they could control and influence the way they were thinking. Some stated that EPIO had provided an opportunity to think differently when in pain and feeling down, as described by one participant:

*The pain is somewhat harmless, it is not a catastrophe, and it will pass. (ID 3)*

Some of the participants also said they had become more aware, and gained a better understanding, of themselves. For example, describing being more aware of, and paying more attention to, their inner dialogue, what they were thinking, and how they were talking to themselves, in some ways realizing that this dialogue had changed from inner criticism to becoming more supportive. One participant described the experienced changes in cognition and awareness as:

*Learning to think differently, (….). Helps a lot with the pain…and the mind. They are connected, you know. You can do this. This is going to be fine. Relax. Breathe deeply. That is what I told myself. (…) and that I could be proud of myself, and so on. (ID 42)*

The participants also stated that EPIO had contributed to greater awareness and better identification of what leads to stress in life, and reported having realized how stress in everyday life could be related to tensions in their own bodies, which in turn could worsen the pain. As one participant stated:

*Yes, well, it has something to do with stress. Indirectly, it is related to pain in a way, and vice versa. (….). It is not always about the pain, you know, but if I'm too stressed and keep tensing my body all the time, I know that eventually, I'll end up in... in pain again. (ID 9)*

Increased awareness of one's body and how calming down and relaxing were possible was also mentioned. As one participant stated:

*And then the fact that taking a timeout was possible, even if standing or sitting. (….) You just need to be present, sort of. I did not think that was possible. (ID 32)*

#### 3.2.2 Sub-theme 2: acceptance

Participants described how using EPIO had induced more acceptance of the pain, and that changing how they were thinking about the pain had been important. This type of acceptance thinking was described as changing their attitude and taking on a more relaxed or optimistic outlook. As one participant stated:

*You shouldn't get stuck if you're troubled with some chronic pain, it's actually possible to learn to live with it. Yes. That you can make everyday life better. Yeah. I have accepted it a bit more (…). Then I do not feel down as much, if you know what I mean. I adapt my everyday life more now, based on how the day is going. (ID 53)*

Other participants described how changing their thought patterns had enabled them to think in a new, more accepting way, allowing them to live better with pain. As one participant said:

*There are other ways of thinking about it. I liked the fact that it (EPIO) made me think in a different way. That you get…well, I liked the psychology. That you get a chance to have new thoughts about your situation, and a chance to live better with pain. (ID 3)*

Further describing acceptance in terms of being able to live with pain, participants also stated that using EPIO helped them understand how accepting pain is not the same as giving up, and that they can live with, and improve, despite having pain. As one of the participants reported:

*Just acknowledging that it (the pain) is there. How much it can do. I never would have thought. Because I thought that if you acknowledge that it is there, it will get worse, but it does not, you know. Acknowledging pain and just letting it go. It was a bit easier than I thought it would be. (ID 32)*

#### 3.2.3 Sub-theme 3: shifting focus

Participants also described shifting focus away from discomfort and negative thoughts toward more positive aspects based on the use of EPIO. This for example included focusing on achieving relaxation when performing relaxation exercises, rather than on how much pain they were experiencing. Some described feeling as if they had more “space in their head” to think about other things besides their discomfort. Others stated that they had more focus on what was realistic for them to achieve, and what was “good enough”. Being able to shift focus depending on the situation was perceived as positive, as expressed by one of the participants:

*It sounds like a dream thing. It is true, you know. I have less focus (on the pain). Maybe I've learned to relax and take breaks a bit, focusing more on that than how painful it can be. The same when I go for a walk with the dogs. If I manage to walk a bit, it is good enough (…), maybe I focus more on what I can do. On what is good enough? (ID 211)*

Having access to EPIO also appeared to have helped participants think more constructively about the pain, as illustrated by one participant:

*Yes, it has helped me not to fall completely into despair. Even though I have had a lot of pain in my body (…). I have been very good at... before... putting myself down. It (EPIO) has helped me a lot, both psychologically and thought wise. Yes, it's about thinking positively and not thinking things about yourself that you would not say to others. Things like that have helped me a lot. (ID 42)*

### 3.3 Changes in coping

Participants described EPIO as having facilitated better coping with pain, subsequently learning how to reduce the pain, particularly when high pain intensity occurred. Having gained tools for improved emotion regulation was also highlighted, with participants describing use of EPIO as inducing hope and providing ways to improve emotional wellbeing. Learning the importance of activity pacing and how to balance activity and rest was also underlined as vital for improved coping and wellbeing. Themes raised were sorted into three sub-themes: Pain, Emotions and Activity Pacing.

#### 3.3.1 Sub-theme 1: pain

Pain was often described as fluctuating from day-to-day, even within days, being severe, uncomfortable and bothersome, creating frustration and worries. Despite this, participants reported having become better able to cope with the pain through the use of EPIO, even learning how to reduce the pain, particularly during pain flares. As stated by one participant:

*These relaxation exercises have enabled me to cope much better with the pain when it is at its worst. (…) Because I can actually reduce it somewhat. Some of these exercises make some of the specific pains I have less. Especially those related to the neck and headaches.... that's where I've gained a lot, actually. (ID 47)*

#### 3.3.2 Sub-theme 2: emotions

Participants also described how EPIO appeared to change and impact emotions by helping them feel less frustrated, ashamed, or guilty. They reported changes in terms of improved ability to deal with negative emotions associated with pain, and described how EPIO had helped prevent emotional setbacks, as well as helped reduce momentary symptoms of stress, anxiety, and depression, even being useful during panic attacks. As one participant reported:

*It (EPIO) has helped me. Because I have been struggling with stress for a while. I got these panic attacks and such. It helped me. Completely unbelievable, really. (ID 42)*

Participants also portrayed having developed a more positive attitude toward life, and stated that they felt happier, had more energy, and that life was not so difficult anymore, perhaps even easier. Some also said that they had gotten their lives back, or that they had gained hope that they could actually get better. As one participant stated:

*I feel like maybe I am a bit more of a happy person (laughs). I was happy before, of course, but you feel, in a way, you now have some tools that help you in everyday life - to cope (...). Then it doesn't feel as difficult, even though, of course, there are difficult days in between, everyone has that. Still, I think things are a bit easier and less dark, you know. (ID 51)*

#### 3.3.3 Sub-theme 3: activity pacing

Some of the participants described a change in their approach to activity since becoming acquainted with EPIO, having become more aware of the necessity to pace themselves, balancing activity and rest, and reported actively working on this. As one participant said:

*I have realized that I need rest. The biggest change is that I have started taking breaks. I've always been very bad at taking breaks. (ID100)*

Participants also described having recognized the importance of making better decisions about what to participate in and not, from the use of EPIO, and having learned to set boundaries for their own activities, without feeling guilty. Describing how this had helped them cope better with challenges in daily life, some of them reported stopping more often, checking in with themselves, and taking more breaks than before. As stated by one of the participants:

*I have always known that there should be a balance between activity and rest, but I have become much more conscious about this and have learned how to handle it. I think about setting boundaries, what I should be involved in and say yes to, and I have become better at saying “no”, it's enough now. (ID 211)*

### 3.4 Content and functionality specific engagement

Having described how use of EPIO had contributed to changes in ways of thinking and coping, participants also highlighted how specific content and functionalities had contributed to these changes, augmenting the engagement and motivation for use of the EPIO intervention program. Identified aspects were sorted into three sub-themes: Breathing and Other Mind-body Exercises, Thought-reflection Exercises, and Functionalities.

#### 3.4.1 Sub-theme 1: breathing and other mind-body exercises

All participants emphasized enjoying and benefiting from the various short- and long breathing (i.e., diaphragmatic breathing) exercises in the EPIO program. They pointed to the breathing exercises as particularly effective, easy to use and remember, and also described them as easy to use without actually accessing the EPIO program, as these exercises could be used anywhere, even in social settings, without anyone noticing. Participants also expressed surprise that slow and deep breathing could have such an immediate and soothing effect on pain, and on emotional comfort. As described by one of them:

*That is what I have focused on, those breathing exercises. I also had (…surgery), and I was lying on the floor here doing breathing exercises (….). And I felt like I got through it somehow....those breathing things have been helpful in many situations. Yes. Breathe with your stomach and relax. (ID 22)*

Other types of EPIO relaxation or focusing exercises (e.g., progressive muscle relaxation, visualization, mindfulness) were also described to be of benefit and fostering use, with participants stating how the exercises helped them fall more easily asleep, achieving better sleep quality, and how using the exercises was pleasant in and of itself, giving them a break in everyday life and a time-out from discomfort. As one participant stated:

*Then one of those visualization exercises helped me change perspective and imagine new and different types of scenarios, helped me reduce the (…) overload in my head. (ID 3)*

Participants also reported having become better at calming down and relaxing in the moment, clearing their minds, and having attained a sense of physical and psychological wellbeing from engaging in the EPIO mind-body exercises. As described by one of the participants:

*Using the guided exercises empties my head completely, and I am just in that moment...And that brings a feeling of wellbeing inside my body. (ID 100)*

#### 3.4.2 Sub-theme 2: thought-reflection exercises

Several of the participants described experiencing the EPIO thought-and-reflection exercises as particularly beneficial, often in combination with EPIO psycho-educational content. They referred to these exercises as helping them recognize the impact of experiences and hassles, to better understand their own approach and reaction to stressors, and to aim to sort out their own thoughts and emotions. As one participant said:

*I have a little bit more clarity, and better follow-up from my doctor because I asked for it. And then you realize that you don't stress as much. First, you must figure out what stresses you out, and then you can clean it up. (ID 32)*

Participants also described thought challenging aspects of EPIO as encouraging contemplation and providing strategies to take action and make specific changes in own life. As one participant stated:

*I can handle daily life now, simply put. Being able to work… I have been at work much more this past year than before. So, I think that the (EPIO) app helped me sort out what is important. (ID 34)*

#### 3.4.3 Sub-theme 3: functionalities

The participants also pointed to the EPIO program design aspects as motivating for use. The voice guiding participants through the program was for example described as friendly and pleasant, providing calmness, speaking to the participants and providing a feeling of being with someone, as if they were friends or had a type of relationship. As one participant said:

*It felt like someone was talking to you. You only heard the voice, quiet and calm. I only had my headset on, so I didn't hear anything else. Just the voice. It was as if you disconnected completely from everything else and just calmed down. (ID 63)*

Some of the participants also highlighted the importance of good explanations related to content, stating that EPIO was easy to understand and practice. The availability of the app, the potential for daily reminders, the fact that exercises can be stopped when desired, and the options for customization according to individual needs, daily condition, and/or location (e.g., through “My page”) were also underlined as promoting engagement and motivation for use.

## 4 Discussion

Interviews identified how people living with chronic pain described experiencing *Changes in Cognition, Changes in Coping*, and *Content and Functionality Specific Engagement* after 12-month access to the EPIO digital pain self-management program. Each of the main themes consisted of sub-themes, including increase in *insight, self-awareness and acceptance*, shift in *focus, pain reduction*, improvements in *emotion regulation*, and a recognition of the importance of *activity pacing*. Content and functionality specific aspects contributing to engagement and motivation for use included exercises such as diaphragmatic *breathing* and *thought-reflection*, and certain program *functionalities*, such as the audio voice and options for personalization, were also described as contributing to use, engagement and change.

The interviews in the current study were conducted after completion of a 12-month RCT showing statistically significant between-group differences in favor of the intervention group (i.e., having access to EPIO for 12 months) for *psychological* variables such as anxiety, depression, self-regulatory fatigue, certain HRQoL sub-scales (i.e., general health, vitality, role emotional and mental health), and pain catastrophizing, but not for more *physically* related variables such as pain interference, pain severity, physical HRQoL sub-scales (e.g., physical functioning, role-physical or bodily pain), or pain acceptance (Solberg Nes et al., 2024). These findings might be explained by the fact that the EPIO program was developed to help people gain knowledge and beneficial coping strategies when living with chronic pain (Ledel Solem et al., 2020; Solberg Nes et al., 2024). Potential limitations in the sensitivity of the instruments used or the need for an even longer duration to observe significant effects could be other possible factors.

### 4.1 Changes in cognition and coping

The EPIO program is primarily CBT-based, with some aspects of ACT (e.g., module 5: “What is important to me”), and rather than nurturing thoughts about a pain-free life, the program is as such designed and developed to strengthen agency, provide knowledge, raise awareness and engage users in evidence-based self-management strategies to live well *with* pain (Ledel Solem et al., 2020; Solberg Nes et al., 2024). This aspiration is supported by the current findings describing increased *insight and self-awareness* after 12-month program access. With participants recognizing associations between their own thoughts and feelings, and how these relate in a variety of situations, findings underline the essence of being conscious of inner experiences and aware of own thoughts, emotions and behavior patterns (for example seeing pain as “not a catastrophe”, and “learning to think differently”), aspects considered essential for positive change to occur (Beck, 2020). These findings are consistent with previous studies demonstrating that emotion regulation and awareness are critical in chronic pain management, including gender-related differences (Diotaiuti et al., 2022). The results also support qualitative input from follow-up phone calls during the 12-month RCT, as well as findings from the EPIO feasibility pilot, both studies identifying raised awareness about own condition through use, and what could be done to better cope with the situation living with pain (Bostrøm et al., 2022; Solberg Nes et al., 2024). Overall, these results underline the essence of being conscious of inner experiences and aware of own thoughts, emotions and behavior patterns.

Insight and self-awareness might also be seen as prerequisites for acceptance, as acceptance of challenging situations often requires insight into own thoughts and feelings. This qualitative study captures descriptions of enhanced *acceptance* of own situation after 12-month program access for people living with chronic pain, underlining the importance of qualitative explorations, as the quantitative RCT failed to detect statistically significant between group findings for pain acceptance (Bostrøm et al., 2023; Solberg Nes et al., 2024) Other qualitative studies exploring impact of pain self-management programs have also shown links between improved insight, awareness and promotion of acceptance (Braverman et al., 2023).

Similarly, while the 12-month RCT failed to detect significant quantitative impact for physical variables such as the primary outcome of pain interference (Solberg Nes et al., 2024), the current qualitative findings indicate improved coping skills and *pain reduction* when dealing with pain, not letting the pain significantly interfere with everyday life, even during pain flares. These findings could potentially also be explained through the reported improvement in insight, self-awareness and acceptance. Self-awareness has for example been associated with pain reduction in Fibromyalgia (Hsu et al., 2010), and other qualitative studies have shown improved acceptance to be associated with improved coping with pain (Braverman et al., 2023), and in turn also better symptom management (Arfuch et al., 2022). Participants in the current study mainly mentioned acceptance in connection with pain and discomfort (e.g., stress and difficult emotions), describing acceptance as being able to let go (e.g., of negativity), and thus making room for new and more constructive ways of thinking. The *shift in focus* described by participants, for example from concerns and negative thoughts about pain to thinking that pain is annoying, but not dangerous and will pass, further supports this notion. Such a change can also illustrate improved psychological flexibility (McCracken and Morley, 2014), here possibly linked to pain reduction and lower pain interference.

The positive impact seen on psychological variables for people having access to the EPIO program in the RCT (Solberg Nes et al., 2024) was further corroborated by the current qualitative findings. Participants described being better able to deal with difficult thoughts and emotions after using EPIO, employing new strategies to stay calmer and more relaxed, even during challenging times. Consequently, they reported improved *emotion regulation*, attributing these improvements to the use of a variety of strategies employed through EPIO. Having recognized the importance of *activity pacing* for pain management was also highlighted in terms of improved coping skills, with participants describing having learned to better understand own needs and cope with day-to-day challenges. This is also in support of previous findings from the EPIO RCT and feasibility studies, underlining raised awareness about the importance of activity pacing (Bostrom et al., 2020; Solberg Nes et al., 2024).

Given the description of improved acceptance, improved coping with pain and reports of subsequent reduction of pain and pain interference in the current findings, it is possible that the outcome measures used to gauge pain interference and severity (Keller et al., 2004) as well as pain acceptance (McCracken et al., 2004) did not capture these measures well in the EPIO 12-month RCT (Solberg Nes et al., 2024). These are however well validated and widely used outcome measures (Keller et al., 2004; McCracken et al., 2004), and even the HRQoL measure employed (Ware Jr and Sherbourne, 1992) revealed differences between impact on psychological and more physical variables (Solberg Nes et al., 2024). A more likely explanation might therefore be that without clear quantitative impact, qualitative measures can complement and further amplify our understanding of quantitative findings, such as in the current study. This notion is supported by the EPIO feasibility studies, where qualitative findings identified improved awareness and acceptance (i.e., “making peace with the presence of pain”) as benefits from engagement with EPIO (Bostrøm et al., 2022) augmenting quantitative feasibility findings (Bostrom et al., 2020).

### 4.2 Content and functionality specific engagement

The combination of CBT/ACT-based psycho-educational content and exercises representing well-known concepts (e.g., diaphragmatic breathing, progressive muscle relaxation, visualization, mindfulness) and functionalities such as a guiding avatar (i.e., the EPIOS bird), options for personalization, and choices to read and/or listen was also explored in terms of engagement, motivation for use and impact on change. Breathing exercises (i.e., diaphragmatic breathing exercises whether brief or long) were particularly highlighted as beneficial and easy to use, with participants describing being able to down-regulate acute pain flares, stress and anxiety in day-to-day life utilizing these exercises. These findings are in support of existing research showing how slow, deep breathing engages the vagus nerve for parasympathetic activation and stress reduction (Russo et al., 2017), potentially also positively impacting symptoms of distress, anxiety and depression (Beng et al., 2016; Cho et al., 2016; Zaccaro et al., 2018) Once learned, the participants also described using these exercises independently from the app. Other types of mind-body exercises such as visualization and mindfulness reportedly assisted with “clearing the mind” and inducing physical calmness, while thought- and reflection type exercises appeared to help sort thoughts and identify stressors, seemingly empowering participants in making lifestyle changes. These findings are in line with descriptions from follow-up phone calls in the RCT describing EPIO as a “useful toolbox” (Solberg Nes et al., 2024). Experiencing how using specific strategies can have an effect may also contribute to strengthened self-control and confidence in own capacity to cope with discomfort, pain and stress, as seen in the RCT through improved self-regulatory capacity after 12 months EPIO program access (Solberg Nes et al., 2024).

Studies have shown therapeutic relationships to be essential for change (Watson, 2016; Watson et al., 2014), potentially having more impact on treatment outcome than the method actually used (Bohart et al., 2002; Elliott et al., 2018; Norcross and Wampold, 2011). While the digital EPIO intervention does not entail a patient-therapist relationship, participants did interact with team member(s) during intervention onboarding (i.e., introductory session), and also received two follow-up phone calls during the first weeks of the intervention. Several of the participants also described the voice guiding them through the program as important, having a calming effect in and of itself. These aspects may indicate a sense of program personalization and support the notion of users considering EPIO “a friend”, as referred to by participants in the current study as well as in the EPIO feasibility pilot (Bostrøm et al., 2022), which again might promote engagement and motivation for use.

General input from interviewed participants pointed to an appreciation of the EPIO program, even though 33% of the participants interviewed had not completed all modules over the 12 study months (see Table 1). Several participants also emphasized that in addition to being a valuable support program in and of itself, the EPIO program could also be of benefit as a supplement to other treatments, whether in combination with a therapist (e.g., physiotherapist or psychologist), or as a follow-up after completing inpatient or outpatient rehabilitation. This is also in line with input received through the follow-up phone calls in the RCT and the feasibility pilot (Bostrøm et al., 2022; Solberg Nes et al., 2024).

### 4.3 System use

The majority of participants in the current study had high module completion (72%), and while participants may have been more likely to agree to being interviewed when having completed the program, program completion percentage for this sample is comparable to that of the full RCT intervention group (66%; Solberg Nes et al., 2024). System use varied (range 5–315 days), with an average (median) use of 37 days. While intended intervention benefit is generally dependent on intervention use and practice, participants did describe utilizing acquired program knowledge (e.g., diaphragmatic breathing exercises) also without accessing the app, which makes gauging actual use more challenging than assessing impact.

### 4.4 Strengths and limitations

This study has a number of strengths. First, the systematically conducted matrix-based recruitment strategy allowed for the study sample to be representative of intervention group participants (e.g., age, gender, pain duration, program use), and presents a major strength. In addition, the qualitative sample included approximately one in four participants completing the 12-month RCT, contributing to the presence of rich, qualitative data complementing quantitative findings (Solberg Nes et al., 2024) and enriching interpretation of findings after 12-month EPIO program access (O'Cathain et al., 2013; Richards et al., 2019).

The current study also has limitations. First, the project staff members who recruited and followed participants during the study also conducted the qualitative interviews. This could have led to participants having a positive approach to the interviews, wanting to please the interviewer. On the other hand, the fact that participants may have felt a connection with the interviewer could have contributed to participants sharing more openly about their experience. The interviewers were also following a semi-structured interview guide and were trained to avoid directing participant responses in one way or other.

Second, study participants were primarily female. However, while the 12-month RCT primarily entailed women (81%), the utilized matrix enabled a 28% inclusion of males, which constitutes an improvement, although not a fully gender balanced participation. No gender-specific patterns were nevertheless detected in the obtained qualitative data. Whether this was due to the limited number of males included, or whether no gender-specific differences were actually present, remains unclear, and future research should aim to further explore gender-related differences as this could provide critical insights for the tailoring of digital pain management interventions. Third, the majority of participants had lived with pain for more than 10 years. Considering this was also the case for participants in the RCT (Solberg Nes et al., 2024), the current study matrix did allow for a fairly representative inclusion regarding participant's pain duration. Fourth, another aspect that might have impacted transferability is the fact that a majority (68%) of participants had completed all program modules and hence could have been more likely to have benefited from the program. This is to be expected, however, as participants completing few modules might have been less willing to participate in interviews. Given the limited information related to participants completing fewer modules, clear conclusions cannot be drawn regarding participants not progressing in the program. Utilizing the matrix, people with lower completion rates were nevertheless also included (please see Table 1), without any clear aspects described as to why program progression was limited or slow.

Fifth, the fact that EPIO RCT recruitment was voluntary (i.e., via health care personnel and social media) could indicate high motivation for participation, another factor potentially limiting transferability, even though motivation is necessary for program utilization and subsequent effect. Sixth, it is also possible that comparison with other digital pain self-management solutions could yield more information as to the reasons for use and resulting change, and future research might consider such comparisons when feasible. Seventh, the current study included people with a wide variety of pain conditions, although the majority with primary musculoskeletal pain (e.g., Fibromyalgia, unspecified musculoskeletal pain). It is possible that tailoring EPIO to more specific pain conditions (e.g., Rheumatoid arthritis or specific post-injury/surgery) could help enhance impact and sense of benefit even further. Previous studies have also shown that factors such as gender and agonistic experience influence pain perception, reinforcing the need for personalized approaches to chronic pain management (Diotaiuti et al., 2022).

Finally, participants being interviewed had to have had completed the 12-month follow-up outcome measures, which could have indicated study engagement, further potentially limiting transferability. Intervention benefit is however dependent on intervention engagement and use, and while the EPIO feasibility pilot sought to identify reasons for use as well as non-use (Bostrom et al., 2020; Bostrøm et al., 2022), the current study strived to identify participants' experiences and achieve a deeper, richer picture of experiences with EPIO than possible from quantitative measures alone. Also, the varying descriptions from the sample interviewed allow for a synthesis example impact from having access to EPIO over a longer period of time (i.e., 12 months). The results can hence contribute to an expanded understanding not only of the changes described from using EPIO, but also of the limitations potentially introduced when solely utilizing quantitative measures in large scale trials such as the EPIO RCT (Solberg Nes et al., 2024).

### 4.5 Conclusion

The current study showed how people with chronic pain, after 12 months access to the EPIO digital pain self-management program, experienced positive changes in terms of living with pain, particularly related to: *Changes in Cognition*, including improved insight, self-awareness and acceptance, and *Changes in Coping*, including pain reduction, improved emotions and wellbeing, as well as increased understanding and use of activity pacing. *Specific Content and Functionalities* appeared to contribute to these changes, creating a sense of connection through program features and facilitating program engagement and motivation for use. The most prominent changes from EPIO program access included an increased understanding of the connection between own thoughts, feelings and behavior, gaining concrete strategies to cope with everyday life living with pain, and utilizing these strategies to reduce pain and the interference of pain, as well as improve psychological wellbeing. A combination of the EPIO program with other clinical interventions such as physical therapy could potentially further enhance outcomes and support widespread adoption.

## Data Availability

Data sets from this study are, due to the nature of patient sensitive information, not available for public sharing through public archives or repositories. Deidentified data from this study will however be made available in accordance with institutional standards through contacting Elin Bolle Strand. Requests to access the datasets should be directed to Lise Solberg Nes, solbli@ous-hf.no.
